# Role of Extracellular Vesicles in Severe Dengue: Virus–Host Interactions and Biomarker Potential

**DOI:** 10.3390/v17060807

**Published:** 2025-05-31

**Authors:** Juan Sebastian Henao Agudelo, Gabriel Pereira, Célio Junior da Costa Fernandes

**Affiliations:** 1 Faculty of Health Sciences, Central Unit of Valle del Cauca (UCEVA), Tulua 763022, Valle del Cauca, Colombia; 2 School of Medicine, Nephrology Division, Federal University of São Paulo, São Paulo 04021-001, Brazil; pereira.gabriel18@unifesp.br; 3 Department of Biophysics and Pharmacology, Institute of Biosciences, Universidade Estadual Paulista “Júlio de Mesquita Filho” (UNESP), Botucatu 18610-307, São Paulo, Brazil; celio.fernandes@unesp.br

**Keywords:** dengue, extracellular vesicles, microRNAs, inflammation

## Abstract

Severe dengue is a global health threat, affecting 4 billion people, with nearly 1 million hospitalizations during epidemics and around 25,000 annual deaths. Severe dengue presentations are characterized by vascular leakage, hemorrhagic manifestations, and shock, which can lead to multiorgan failure. Recent studies highlight the crucial role of extracellular vesicles (EVs) in the pathogenesis of dengue, influencing immune response and disease progression. EVs, nanometric structures secreted by cells, mediate viral dissemination, immune modulation, and endothelial dysfunction by transporting biomolecules such as microRNAs (miRNAs) and viral proteins. Infected cell-derived EVs carry viral components, including NS protein and miRNAs like miR-21 and miR-126-5p, which compromise endothelial integrity and activate immune pathways such as Toll-like receptor, NF-κB, and JAK-STAT signaling. This, together with the immune response, leads to the release of pro-inflammatory cytokines, including TNF-α, IL-1β, IL-6, and IFN-γ. EVs also facilitate viral immune evasion by suppressing antiviral responses. Recent analyses of miRNAs within EVs suggest their potential as biomarkers for disease progression. Differentially expressed miRNAs in circulating EVs correlate with severe outcomes, providing tools for risk stratification and therapeutic monitoring. Advanced techniques, such as nanoparticle tracking analysis and flow cytometry, allow precise EV characterization, supporting their integration into clinical applications.

## 1. Introduction

Dengue is an acute viral disease caused by one of the four dengue virus serotypes (DENV-1, DENV-2, DENV-3, and DENV-4), transmitted to humans through the bite of mosquitoes of the genus *Aedes*, primarily *Aedes aegypti* [1]. Factors such as unplanned urbanization, human migration, and climate change have facilitated the spread of both the vector and the virus [2]. Annually, it is estimated that 390 million infections occur across 129 countries, with approximately four billion people at risk [3]. The regions most affected by dengue include Latin America and the Caribbean, Southeast Asia, and parts of tropical Africa, where *Aedes aegypti* is endemic. Brazil, India, and Indonesia report the highest global case numbers, with recurrent outbreaks in countries such as Colombia, Mexico, the Philippines, and Thailand [4]. Between 75% and 80% of those infected are asymptomatic, suggesting significant underreporting of cases. During epidemic peaks, nearly one million individuals require hospitalization, and severe forms of the disease cause approximately 25,000 deaths annually, with case fatality rates ranging from 1% to 5% [4]. In response to the exponential increase in cases over the past two decades, the World Health Organization (WHO) declared dengue a Level 3 emergency in 2023 [5].

Dengue exhibits a wide range of clinical manifestations and can rapidly progress from mild febrile illness to severe and life-threatening forms [6]. While most infections are self-limiting and present as dengue fever, a significant proportion can advance to severe complications, such as dengue hemorrhagic fever (DHF) or dengue shock syndrome (DSS) [6]. These severe forms are often associated with multi-organ failure and high mortality rates [7]. The unpredictable and dynamic nature of the disease, coupled with its growing global incidence, underscores the urgent need to strengthen surveillance, prevention, and clinical management strategies, particularly in highly affected regions [7].

Despite significant research, the immunopathological mechanisms underlying the development of DHF remain incompletely understood. It has been proposed that DHF may result from dysregulated lymphocytic and humoral responses, associated with phenomena such as immunologic imprinting and antibody-dependent enhancement (ADE) [8]. These mechanisms may explain both the clinical severity and the increased vascular permeability characteristic of DHF in patients experiencing secondary infections [8]. Additionally, the cytokine storm triggered by macrophages, dendritic cells, and mast cells appears to be a key driver of plasma leakage [8]. Previous studies have shown that the virus does not directly cause endothelial damage; instead, cytokines such as Tumor necrosis factor-alpha (TNF-α), and interleukins (IL-1β, and IL-6) contribute to thrombocytopenia and immune hyperactivation. These cytokines, together with non-structural protein 1 (NS1) immune complexes, induce endothelial dysfunction and promote vascular permeability, suggesting that the inflammatory microenvironment and paracrine processes play central roles in severe dengue pathophysiology [9].

A recently highlighted paracrine mediators in dengue is extracellular vesicles (EVs), which have emerged as critical players in dengue pathogenesis, contributing to immune modulation, endothelial damage, and viral transmission [10]. EVs are small membrane-bound structures released by eukaryotic and prokaryotic cells in response to stimuli such as cellular stress and apoptosis [1]. These vesicles play crucial roles in intercellular communication, molecular transport, and immune system modulation, and they can be detected in body fluids, underscoring their potential as biomarkers [1].

In this context, this review aims to synthesize and analyze the latest research on the role of EVs in dengue pathogenesis. Specifically, it will address their ability to elucidate the physiological mechanisms underlying vascular permeability, plasma leakage, and immune evasion. Additionally, their potential impact on the development of clinically relevant biomarkers such as microRNAs contained within EVs will be explored, emphasizing on their use for patient monitoring and clinical classification through advanced bioinformatics tools. Finally, potential therapeutic strategies based on current knowledge of EVs will be discussed, highlighting their significance in managing severe dengue.

## 2. Materials and Methods

### 2.1. Search Strategy

**Figure 1:**
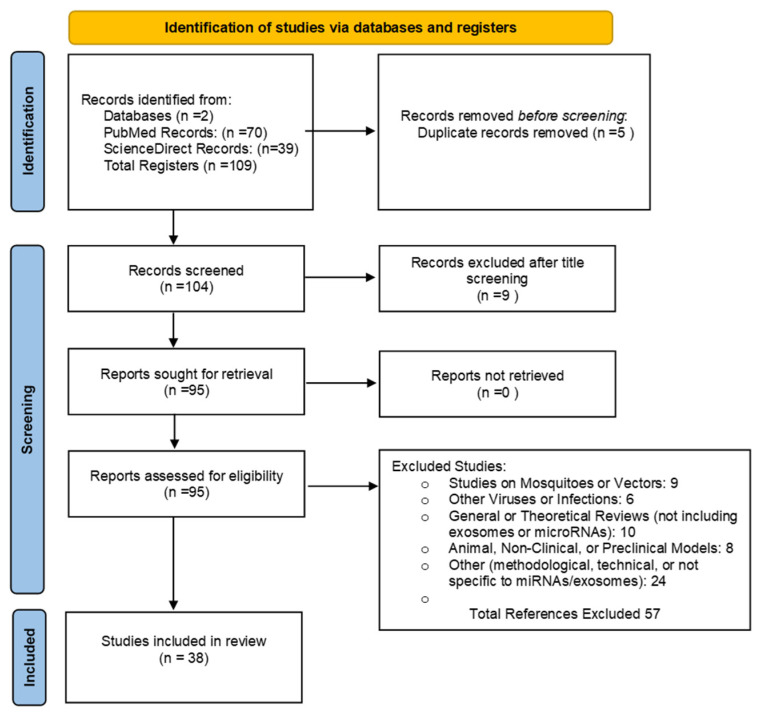
Representation of the PRISMA flow diagram depicting the steps involved in identifying, screening, and selecting articles for inclusion in the review.

This study employs a narrative review to analyze recent research on the role of extracellular vesicles (EVs) in the pathogenesis of severe dengue. The review aims to address the following research question: What is the role of extracellular vesicles (EVs) in dengue pathogenesis, and how can they contribute to understanding vascular permeability, plasma leakage, immune evasion, and the development of clinically relevant biomarkers and therapeutic strategies? To robustly address this question using relevant and up-to-date evidence, we conducted a comprehensive literature search in two databases, ScienceDirect and PubMed, following the guidelines of the Preferred Reporting Items for Systematic Reviews and Meta-Analyses (PRISMA) framework [11]. The search strategy was based on the following equation: (‘Extracellular Vesicles’ OR ‘Exosomes’ OR ‘Microvesicles’ OR ‘microRNAs’) AND (‘Dengue’ OR ‘Severe Dengue’). To enhance the sensitivity and specificity of the search, we incorporated Medical Subject Headings (MeSH) terms. The detailed methodology is illustrated in Figure 1. Additionally, to update data related to the epidemiology and general characteristics of the viral infection, the following search terms were used: (‘Dengue’ OR ‘Severe Dengue’ OR ‘Dengue Hemorrhagic Fever’) AND (‘Epidemiology’).

### 2.2. Eligibility Criteria

The inclusion criteria for this review focused on peer reviewed journal articles, review articles, research papers, and book chapters published in English, Portuguese, or Spanish, within the last 10 years (2014 to 2024), that involved human studies or preclinical research. Articles were excluded if they primarily addressed mosquitoes or vectors without discussing extracellular vesicles (EVs) or microRNAs (miRNAs) in the context of dengue, investigated viruses or infections unrelated to dengue, were general or theoretical reviews that did not specifically cover exosomes, EVs, or miRNAs in relation to dengue pathogenesis, involved animal models or non-clinical studies without direct relevance to human clinical data on dengue, or were methodological or technical papers not focused on miRNAs, exosomes, or EVs, lacking clinical applicability to dengue. To ensure the quality of the paper, duplicates were meticulously reviewed, and article abstracts were included to verify the relevance and academic integrity of the literature

### 2.3. Bioinformatic Analysis of miRNAs in Dengue

Bioinformatics analyses were conducted to investigate the relationship between circulating microRNAs (miRNAs) within EVs and dengue virus (DENV) infection or disease severity. A reanalysis of differentially expressed miRNAs (DEmiRs) was performed using the RNA sequencing dataset GSE150623, obtained from the Gene Expression Omnibus (GEO). This dataset represents the circulating microtranscriptome of dengue patients. The analysis was carried out using the limma voom [12] pipeline in R Studio version 4.3.2 [13], enabling the identification of miRNAs potentially associated with disease progression and severity. DEmiRs cutoffs were set as |LogFC| > 2 and adjusted *p*-value < 0.05. Enrichment analysis of target genes of DemiRs was performed through Diana miRPath v4 [14].

## 3. Clinical Presentation and Structural Overview of the Dengue Virus

The dengue virus is an enveloped 50 nm virus belonging to the Flavivirus genus and the Flaviviridae family [15]. Its structure is icosahedral, and its genome consists of a single-stranded positive-sense RNA. This genome encodes a single open reading frame (ORF), which is translated into a polyprotein [15]. The polyprotein is subsequently processed into three structural proteins (capsid [C], envelope [E], and prM/M) and seven nonstructural proteins (NS1, NS2A, NS2B, NS3, NS4A, NS4B, and NS5). These proteins play critical roles in viral assembly, replication, and immune evasion within the host [15].

To date, four clinically relevant dengue virus serotypes have been identified: DENV-1, DENV-2, DENV-3, and DENV-4 [16]. These serotypes are antigenically related, sharing 60–75% amino acid similarity. This limited cross-protective immunity contrasts with other viral infections. Within each serotype, viruses can be further divided into genotypes, differing genetically by up to 6% [16]. These genetic variations can significantly influence virulence, disease severity, and the epidemiological dynamics of outbreaks [15,16].

Dengue pathogenesis progresses through three distinct phases: febrile, critical, and recovery, which define the clinical course of the disease. According to the 2009 WHO classification, dengue is categorized into dengue without warning signs, dengue with warning signs, and severe dengue. The febrile phase begins after an incubation period of 2 to 7 days, characterized by sudden fever exceeding 38 °C, accompanied by symptoms such as headache, retro-orbital pain, myalgia, nausea, and rash [17]. During this stage, viremia is high and detectable via quantitative polymerase chain reaction (qPCR), peaking within the first 4–5 days [17]. Common complications include dehydration and febrile seizures in children due to high fever. In most cases, the infection is mild and self-limiting [17].

The critical phase, occurring in some patients, particularly during heterotypic reinfections, lasts 24–48 h and coincides with declining viremia and fever. This stage carries a higher risk of complications due to an exaggerated inflammatory response causing endothelial damage and increased capillary permeability [18]. This can lead to warning signs such as abdominal pain, persistent vomiting, bleeding, and hepatomegaly. Severe cases may progress to significant fluid leakage, shock, and organ dysfunction affecting the liver, kidneys, and central nervous system (CNS) [18].

Finally, the recovery phase involves the reabsorption of fluids and clinical stabilization. However, some patients may experience post-infection symptoms, including fatigue, muscle pain, depression, and neurological complications, particularly in adults [19].

## 4. Immune Response and the Potential Role of EVs in Dengue Severity

**Figure 2:**
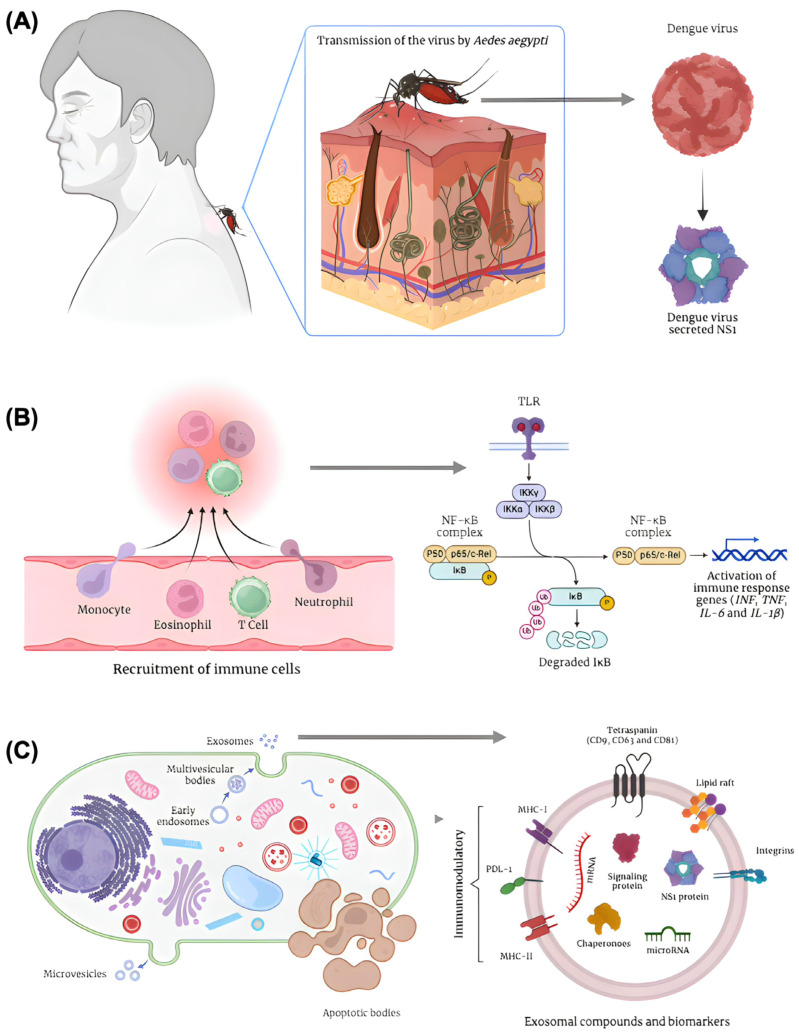
Impact of dengue virus infection—Role of the NS1 Protein, Immune Signaling, and Extracellular Vesicles: (**A**) Dengue transmission occurs through the bite of the Aedes aegypti mosquito, the vector of the dengue virus, a positive-sense single-stranded RNA virus belonging to the genus Flavivirus. The virus encodes the non-structural protein 1 (NS1), a multifunctional glycoprotein essential for viral replication and pathogenesis, contributing to endothelial dysfunction and immune modulation. (**B**) Following viral infection, a signaling cascade is activated, involving the recruitment of immune cells and increased vascular permeability. This response induces cytoskeletal alterations, oxidative stress, and inflammatory signaling mediated by the activation of Toll-like receptors (TLRs). These receptors ultimately lead to the activation of the inflammatory pathway regulated by nuclear factor kappa B (NF-κB), promoting the immune response and the transcription of related genes such as interferons (INF), tumor necrosis factor (TNF), and interleukins (IL-6 and IL-1β). (**C**) Immunologically active cells may undergo apoptosis, releasing apoptotic bodies, or activate paracrine signaling pathways mediated by extracellular vesicles, including exosomes. These exosomes transport RNA, microRNAs, and the viral NS1 protein itself, playing an immunomodulatory role through receptors such as MHC-I, MHC-II, and PDL-1. These mechanisms contribute to the propagation of inflammatory signaling and the modulation of the immune response during dengue virus infection. Figure created using BioRender.com.

Dengue infection begins with the mosquito bite, introducing the virus into the dermis (Figure 2A). There, Langerhans cells recognize the virus through pattern recognition receptors (PRRs) such as Toll-like receptors (TLRs), RIG-I, and MDA5 [20]. This triggers the production of type I interferons (IFN)-α/β by macrophages, epithelial cells, and dendritic cells (DCs). The DCs migrate to lymph nodes, where they facilitate viral dissemination to monocytes and other DCs, amplifying viremia and targeting organs such as the liver, spleen, and vascular endothelium [20].

The innate immune response, led by macrophages, DCs, and natural killer (NK) cells, produces pro-inflammatory cytokines such as IL-1, IL-6, IL-8, and TNF-α, responsible for early symptoms like fever and malaise (Figure 2B) [21]. Concurrently, DCs present viral antigens to T and B lymphocytes, activating the adaptive immune response. CD8+ T lymphocytes eliminate infected cells, while CD4+ T cells adopt a Th1 phenotype, promoting viral clearance via IFN-γ production B lymphocytes generate antibodies targeting structural and nonstructural viral proteins, including M, E, and NS1. This adaptive response clears viremia and establishes specific immune memory, mediated by protective IgG antibodies against reinfection by the same serotype [21].

Severe manifestations often arise during heterotypic secondary infections. In these cases, pre-existing IgG antibodies fail to efficiently neutralize the new serotype, facilitating viral entry into monocytes and macrophages via Fc receptors. This process, known as antibody-dependent enhancement (ADE), increases viral replication and inflammation, contributing to disease severity [22]. ADE excessively activates innate immune cells, triggering the release of cytokines such as TNF-α, IL-1β, IL-6, and IFN-γ, along with vasoactive mediators like histamine and tryptase released by mast cells. These factors cause endothelial damage and heightened vascular permeability, resulting in plasma leakage characteristic of severe cases. CD8+ T lymphocytes activated during secondary infections further exacerbate inflammation, producing excessive pro-inflammatory cytokines due to their low affinity for the new serotype [22]. However, ADE or immunologic imprinting can explain not all severe dengue cases. Up to 5% of patients may develop severe forms during primary infection, suggesting that other factors such as host genetics, age, and viral genotype also contribute to disease severity [22].

Extracellular vesicles have garnered significant attention in the study of dengue pathogenesis, particularly for their multifaceted roles in disease progression [10]. As dynamic carriers of biomolecules, EVs derived from infected patients and in vitro models have been implicated in exacerbating dengue severity, including plasma leakage and endothelial dysfunction. Their ability to mediate immune interactions positions them as pivotal agents not only in dengue but also in other viral infections such as hepatitis, HIV, and flaviviruses [23].

In severe dengue, EVs contribute to pathophysiological processes by facilitating viral dissemination, disrupting vascular integrity, and modulating host immune responses. Investigating EVs provides valuable insights into the molecular mechanisms driving these outcomes and offers potential pathways for developing advanced diagnostic tools and therapeutic interventions tailored to mitigate the impacts of severe dengue [10,23].

## 5. Biogenesis of Extracellular Vesicles and Their Role as microRNA Carriers in Dengue Virus Infection

Extracellular vesicles (EVs) are nanometer-sized structures released by nearly all cell types and play a central role in intercellular communication. Encapsulated by lipid bilayers, these vesicles carry bioactive molecules, including RNA, proteins, and lipids, which can modulate physiological and pathological processes in recipient cells. The synthesis of EVs involves different molecular pathways, depending on the specific vesicle subtype [24]. The diversity of these biogenesis mechanisms reflects the functional heterogeneity of EVs and their adaptability to cellular contexts [25,26]. The molecular machinery that regulates the biogenesis of these vesicles not only determines their formation but also influences the selection of content, membrane composition, and, ultimately, their biological effects on recipient cells [27].

Apoptotic bodies, for example, are formed during programmed cell death. As apoptosis progresses, the activation of caspases triggers the cleavage of cytoskeletal components, resulting in cell fragmentation [28]. Segments of the plasma membrane, forming apoptotic bodies (Figure 2C), enclose these fragments. Unlike other EVs, apoptotic bodies can encapsulate organelles, chromatin fragments, and other cellular debris, serving as a mechanism to remove dead cells and modulate immune responses [29,30]. On the other hand, microvesicle formation occurs through the budding of the plasma membrane, a process driven by cytoskeletal rearrangements. The biogenesis of these vesicles depends on changes in membrane lipid asymmetry, mediated by enzymes such as flippases, floppases, and scramblases [31,32]. The activation of these enzymes promotes the externalization of phosphatidylserine, contributing to membrane curvature and budding. Actomyosin contractions, regulated by Rho-associated kinase (ROCK) and calcium signaling, provide the mechanical force needed for membrane fission [33,34]. Microvesicles are often enriched with integrins, selectins, and metalloproteinases, which contribute to their functional specialization and targeting capability (Figure 2C).

Exosomes, distinct from apoptotic bodies and microvesicles, are formed within multivesicular bodies (MVBs) through either ESCRT-dependent or ESCRT-independent mechanisms [33,35]. Within MVBs, intraluminal vesicles (ILVs) form inside late endosomes. The ESCRT machinery, composed of four main complexes (ESCRT-0, -I, -II, and -III), coordinates this process [35]. ESCRT-0 recruits ubiquitinated cargo to the endosomal membrane, while ESCRT-I and -II promote membrane deformation, and ESCRT-III carries out membrane cleavage. Accessory proteins such as ALIX (ALG-2-interacting protein) and TSG101 (tumor susceptibility gene 101) interact with cargo and ESCRT components to fine-tune the vesicle formation process (Figure 2C). Exosome biogenesis can also occur via ESCRT-independent pathways, in which lipids like ceramides and tetraspanins (e.g., CD9, CD63, and CD81) play critical roles in membrane curvature and vesicle formation [35]. After formation, MVBs are transported along microtubules, guided by motor proteins like kinesins and dyneins, to the plasma membrane [36]. Rab family proteins, especially Rab27a and Rab27b, regulate MVB anchoring and fusion with the plasma membrane, resulting in exosome release into the extracellular space [36,37].

In this context, EVs are crucial vehicles in intercellular communication, mediating the efficient transport of microRNAs (miRNAs) and other biomolecules, particularly during viral infections [38,39]. miRNAs carried by EVs play essential roles in modulating host responses and promoting viral pathogenesis, acting as post-transcriptional regulators of genes involved in viral replication, immune response, and inflammation [40]. In the context of dengue virus (DENV) infection, studies show that EVs released by infected cells carry specific miRNAs that can significantly alter infection dynamics and virus–host interactions [41].

During DENV infections, exosomes can carry miRNAs such as miR-21, miR-146a, and miR-155, which play crucial roles in modulating innate immunity [42,43]. For example, miR-21, often enriched in EVs during viral infections, negatively regulates genes involved in apoptosis control and viral replication, promoting cell survival and allowing continuous viral replication [44]. miR-146a suppresses pro-inflammatory pathways, such as nuclear factor kappa B (NF-κB) signaling, reducing excessive immune responses and promoting viral persistence [45]. miR-155, in turn, is associated with the amplification of the immune response, being a miRNA with potentially ambiguous effects depending on the stage of infection. In addition to host-derived miRNAs, EVs can also carry viral RNA. Studies have demonstrated that EVs released during DENV infections contain fragments of viral genomic RNA [42,45,46]. These EVs can facilitate viral spread to adjacent cells, contributing to infection amplification as well as modulation of the immune microenvironment (Figure 2C).

Recent studies have confirmed the presence of specific miRNAs in circulating EVs during DENV infection. Proteomic and transcriptomic analysis revealed that exosomes derived from dendritic cells infected with DENV carry miRNAs such as miR-21-5p, miR-222-3p, and miR-126-5p, which play important roles in regulating immune and inflammatory responses [47,48]. For example, miR-126-5p is involved in maintaining vascular integrity, and its dysfunction mediated by DENV contributes to the increased vascular permeability observed in severe dengue [49,50]. Additionally, miR-222-3p has been associated with the suppression of antiviral responses by negatively regulating genes such as STAT3 and SOCS3, critical components of interferon signaling [50].

Exosomes released by infected cells have also been described as modulators of macrophage activation, promoting a pro-inflammatory phenotype that exacerbates the immune response and contributes to dengue pathogenesis. The transport of specific miRNAs to immune cells via EVs illustrates the ability of these vesicles to reshape the immune microenvironment, promoting both viral dissemination and exacerbated inflammation.

## 6. Overview of EVs’ Role in Dengue Infection: Transmission Dynamics

During DENV infection, infected cells release EVs enriched with viral genetic material, including complete genomic RNA and structural and non-structural proteins like NS1 and E [41]. These vesicles can act as vectors to infect neighboring cells, as the viral content within the EVs is protected from extracellular enzymatic degradation. Recent studies show that EVs can promote the entry of viral RNA into uninfected cells, where the viral replication cycle is initiated, contributing to the amplification of viral load in the host [47,51]. This process demonstrates that EVs are not merely a byproduct of the infection but active participants in viral dissemination.

Additionally, EVs can influence viral tropism. For example, exosomes released by infected hepatocytes may contain specific membrane proteins that facilitate fusion with target cells, such as monocytes and endothelial cells [38,52]. This specificity in targeting can enhance infection efficiency in critical tissues, such as the liver and blood vessels, exacerbating disease progression and contributing to the systemic spread of the virus [53,54].

Besides transporting viral components, EVs derived from infected cells play a central role in modulating immune responses. They can carry immunomodulatory molecules that suppress or redirect host responses to favor viral replication. For example, viral proteins carried in EVs, like NS1, can inhibit the IFN pathway in target cells, weakening the innate antiviral response [55,56]. This mechanism allows DENV to evade early detection by the immune system, facilitating its replication and spread. Furthermore, EVs containing specific microRNAs can directly influence cellular processes in the host [57]. The transport of miRNAs such as miR-146a and miR-21, widely described in viral infections, has been associated with the suppression of inflammatory pathways and regulation of the immune environment [58]. These miRNAs modulate the expression of genes crucial to the immune response, creating a cellular microenvironment conducive to viral replication and spread to other cells [38,59].

Extracellular vesicles are also implicated in regulating viral load and clinical progression of DENV infection. Studies show that elevated levels of EVs circulating in association with dengue virus (DENV) infection correlate with higher viral loads and greater disease severity [38]. This association can be attributed to the role of EVs in local infection amplification, transferring viral material directly to neighboring cells and modulating immune responses in ways that avoid efficient viral clearance [38,47].

Moreover, EVs derived from infected cells can alter the function of immune cells such as macrophages and dendritic cells, promoting the secretion of pro-inflammatory cytokines like IL-6 and TNF-α [60]. This increase in cytokine production can trigger systemic inflammatory responses, contributing to plasma leakage syndrome and other severe symptoms of dengue, such as hemorrhagic fever and shock.

## 7. Extracellular Vesicles as Mediators of Viral Spread and Immune Evasion in Dengue Virus Infection

Recent evidence suggests that extracellular vesicles (EVs) present in dengue patients, as well as those derived from virus-infected innate immune cells, have the ability to modulate the immune response, induce inflammation, and, in some cases, transport the viral genome. These EVs may influence dengue pathogenesis by facilitating viral spread and altering immune system function.

On the other hand, it has been demonstrated that EVs released by virus-activated platelets contain IL-1β and TNF-α, which together contribute to inflammation and play a central role in endothelial activation and plasma leakage during severe dengue [61]. Along the same lines, platelets from dengue patients have been shown to release EVs that stimulate the expression of inflammatory markers such as CRP, SAA, sVCAM-1, and sICAM-1 in endothelial cells, thereby promoting leukocyte adhesion, activation, and endothelial inflammation [62]. Moreover, the microRNA miR-96-5p, identified in patient-derived exosomes, has been found to trigger the IL-1β inflammatory pathway by downregulating HSP70, a natural inhibitor of the NLRP3 inflammasome [63]. These findings further support the role of microRNAs as key mediators of inflammation in dengue.

According to Kumari et al., EVs derived from patients with severe dengue express PD-L1 on their surface. This protein interacts with CD4+ lymphocytes, inhibiting their proliferation and increasing the expression of the PD-1 coreceptor [64]. Furthermore, these EVs carry pro-inflammatory cytokines such as IFN-γ, TNF-α, IL-2, IL-6, IL-17A, IL-13, IL-5, and IL-4, which appear to contribute both to inflammation and the anergy observed in the study [64]. Similarly, another study demonstrated that dendritic cells (DCs) infected with the dengue virus can secrete EVs containing mRNA of inflammatory mediators such as CXCR4, MIF, IL-17A, IL-8, and IL-6, which could be translated and exacerbate inflammation [47].

Additionally, Mishra et al. showed that EVs derived from dengue-infected monocytes can transfer miR-148a, modulating USP33 and ATF3 levels in human microglial cells [65]. This process may contribute to the expression of TNF-α, NF-κB, and IFN-β, factors associated with neuroinflammation—a complication observed in severe dengue cases that remains poorly understood from a pathophysiological perspective [66]. These findings suggest that EVs play a significant role in dengue immunopathogenesis, acting as vehicles that transport molecules capable of modulating the immune response and potentially exacerbating disease-associated complications.

Although further evidence is needed, a potential immune evasion mechanism employed by the dengue virus involves the use of EVs to transport viral RNA or proteins that promote viral replication in target cells [23,47]. In a study led by Martins and collaborators, EVs derived from dengue-infected dendritic cells were shown to infect C6/36 cells in vitro, suggesting that these EVs may carry the complete viral genome [47]. This mechanism could allow the virus to evade detection by antibodies and immune cells such as CD8+ lymphocytes and NK cells, which would typically recognize and eliminate the virus [22].

Moreover, another study demonstrated that EVs derived from Aedes aegypti cells infected with dengue contain proteins such as AAEL017301 (elongation factor-1 alpha) and AAEL002675 (a protein of unknown function) that appear to increase susceptibility to infection in target cells [66]. These findings highlight the critical role of EVs in dengue pathogenesis, emphasizing their potential as modulators of immune responses, facilitators of viral dissemination, and contributors to disease severity. Understanding the mechanisms underlying these processes could pave the way for novel diagnostic and therapeutic strategies.

## 8. The Role of Extracellular Vesicles in Endothelial Damage and Vascular Hyperpermeability in Dengue

The integrity of the endothelial barrier plays a critical role in vascular homeostasis and is severely compromised during dengue infections, particularly in severe cases such as dengue hemorrhagic fever and dengue shock syndrome [67,68,69]. In this context, EVs are emerging as important mediators in processes that lead to endothelial dysfunction, directly contributing to vascular hyperpermeability and plasma leakage [70,71]. Detailed studies of these mechanisms have shed light on how EVs act as vehicles for bioactive molecules, including viral proteins, cytokines, and microRNAs, which alter the behavior of endothelial cells and exacerbate the clinical symptoms of the disease [72,73].

During dengue virus (DENV) infection, infected cells release EVs enriched with viral proteins, such as NS1 and E, that can directly interact with endothelial cells [41]. NS1, in particular, is widely recognized for inducing the degradation of cell junction proteins, such as VE-cadherin and claudins, which are essential for maintaining vascular barrier integrity [41,72]. When carried by EVs, NS1 can reach distant endothelial cells, promoting systemic damage to the vascular network. This effect is not limited to localized areas of infection but extends to peripheral tissues, exacerbating plasma leakage and contributing to severe manifestations of dengue, such as edema and shock [41,73].

Additionally, exposure of endothelial cells to EVs derived from DENV-infected cells triggers metabolic and cytoskeletal changes in these cells [38]. These changes, mediated by proteins and miRNAs transported by EVs, alter actin dynamics and impair the formation of stable cell contacts, further amplifying vascular permeability [74]. EVs also carry molecules that activate inflammatory signaling pathways in endothelial cells, such as the TLR and tumor necrosis factor (TNF) pathways. For example, EVs containing viral RNA can interact with TLRs on the surface of endothelial cells, triggering a signaling cascade that results in the release of pro-inflammatory cytokines, such as IL-6 and TNF-α [74]. These cytokines, in turn, increase the expression of adhesion molecules, such as ICAM-1 and VCAM-1, facilitating leukocyte recruitment and perpetuating inflammation [75]. This continuous inflammatory cycle further aggravates endothelial dysfunction and promotes fluid leakage.

As previously discussed, EVs influence the activity of pathways associated with oxidative stress, such as the generation of reactive oxygen species (ROS) in endothelial cells [76]. This oxidative stress damages cellular components and amplifies endothelial barrier disruption, contributing to the extensive damage observed in severe DENV infections [77].

DENV infection in endothelial cells leads to ROS production through several pathways, including the activation of NADPH oxidase (NOX) and mitochondrial dysfunction. Inhibition of ROS production associated with NOX decreases viral replication, cell death, and secretion of inflammatory cytokines such as IL-6, IL-8, and CCL5 [78].

Additionally, oxidative stress induced by DENV activates signaling pathways that lead to the degradation of the transcription factor Nrf2, which is crucial for the antioxidant response [79]. The degradation of Nrf2 compromises the cell’s ability to regulate oxidative stress, exacerbating inflammation and apoptosis [80].

Autophagy activation, associated with endoplasmic reticulum stress, also contributes to ROS production during DENV infection. This process can have dual effects, facilitating viral replication and protecting against cell death, but also exacerbating oxidative stress [79].

## 9. Role of Extracellular Vesicle-Derived microRNAs in Dengue Severity

EVs are crucial mediators of intercellular communication and play an important role in modulating host–pathogen interactions. In the context of dengue infection, specific miRNAs derived from EVs have been implicated in influencing disease outcomes and response [63,65].

miRNAs are small non-coding RNA molecules, typically 20–22 nucleotides in length, that play a crucial role in regulating gene expression at the post-transcriptional level. Each miRNA can bind to multiple mRNA sequences with varying degrees of complementarity, enabling the simultaneous regulation of hundreds of genes in response to environmental changes.

Plasma EVs are derived from adaptive and innate immune cells as well as non-immune blood cells such as platelets [81]. DENV infection leads to increased release of EVs from severe dengue patients platelets and monocytes [64,65], while also being able to change the load of such vesicles [64].

In search for specific biomarkers candidates of severity of the pathological outcome, many studies aimed at miRNAs as potential biomarkers. Studies often reach different miRNAs candidates as they are usually performed on specific populations, geographically or age-restricted. Still, correlations of differentially expressed miRNAs (DEmiRs) found in DENV infection and disease severity are reported in the literature for circulating miRNAs [82,83], thus indicating the possibility of miRNAs to function as disease biomarkers.

In this context, miRNAs derived from EVs are crucial in modulating immune responses, inflammation, and vascular dysfunction, particularly in the context of severe dengue. By targeting specific mRNAs, miRNAs regulate key pathways involved in these processes, influencing disease progression and severity.

A study showed that DENV-infected cells increase the EVs release and the released levels of miR-148a, which suppresses USP33 and stabilizes the ATF3 protein levels, reporting a crucial role of the miR-148a/USP33/ATF3 axis on pro-inflammatory response and DENV replication maintained by EVs [65]. miR-1204 and miR-491-5p are also reported to influence the DENV replication response through p53 regulation of apoptosis [84].

In the context of vascular dysfunction, miRNAs regulate endothelial cell integrity and permeability, two critical factors in dengue pathogenesis. For example, the regulation of EZH2 expression through miR-150 is suggested to interfere in plasma leakage during DENV infection [85]. miRNAs are known to target junctional proteins and signaling molecules that maintain endothelial barrier function [86,87]. Their dysregulation can lead to increased vascular permeability, plasma leakage, and hemorrhagic manifestations characteristic of severe dengue, as is the case for the DENV-associated endothelial dysfunction related to miR-126-5p [49,50], although the link between miRNAs and thrombocytopenia in DENV is still unknown [82]. Overall, miRNAs derived from EVs represent key molecular players that bridge immune dysregulation, inflammation, and vascular compromise in severe dengue.

## 10. Clinical Applications of EVs in Dengue Diagnosis

Recent research has highlighted the potential of EVs as key biomarkers for the identification and monitoring of severe dengue. Various studies have demonstrated that circulating EVs play a crucial role in the pathological processes associated with this disease [10]. Both their concentration and the presence of specific surface antigens or miRNAs have been identified as valuable tools for diagnosing severe forms of dengue [10].

For instance, Punyadee et al. (2015) reported significantly elevated levels of EVs in patients with DHF compared to those with non-severe dengue or healthy individuals. Moreover, they found that the quantity of these vesicles directly correlated with disease severity [88]. Similarly, Kumari et al. (2023) identified an increase in platelet-derived EVs in patients with severe dengue, underscoring the immunoregulatory role of these vesicles [64].

These findings are consistent with studies in other viral pathologies and even in cancer patients, where an increase in circulating EV concentration has also been observed [89]. In dengue, circulating EVs may also transport viral components, such as the E, C, and NS1 proteins, potentially contributing to viral dissemination [23].

Additionally, EVs derived from patients with severe dengue have shown the ability to carry the immune checkpoint PD-L1, highlighting their immunoregulatory role and potential to induce immune anergy [64]. This phenomenon parallels observations in patients with severe COVID-19, where PD-L1 has been detected in both EVs and circulating monocytes [90], suggesting shared features in immune modulation between the two pathologies.

Recent studies have also identified the presence of specific microRNAs in EVs found in the serum of patients with severe dengue [10]. These microRNAs, which will be discussed in detail later, show promising potential as tools to differentiate between severe dengue, dengue with warning signs, and dengue without warning signs.

## 11. Isolation and Detection of EVs from Dengue Patients

EVs from dengue patients must first be isolated to enable subsequent quantification, phenotypic characterization, and molecular content analysis. These vesicles can be obtained from serum or plasma samples collected with anticoagulants such as EDTA or citrate. Samples may be processed immediately or stored under ultracold conditions for later analysis [91].

Commonly used EV isolation methods include ultracentrifugation, immunoaffinity capture, precipitation, and size-exclusion chromatography [92]. The choice of method should be guided by the specific objectives of the study and take into account factors such as EV yield, purity, and the intended downstream applications, including functional assays [92]. Once isolated, EVs can be characterized using various analytical techniques.

Nanoparticle Tracking Analysis (NTA) is widely employed to assess the size distribution and concentration of EVs. When coupled with fluorescently labeled antibodies, NTA also enables the detection of surface antigens, making it a useful tool for initial phenotypic profiling, particularly in complex biological matrices. However, NTA faces several limitations. One major constraint is the limited number of fluorescence channels, which hinders the simultaneous detection of multiple markers and limits its ability to perform deep immunophenotyping comparable to high-resolution flow or mass cytometry. Additionally, fluorescent NTA may lack the sensitivity required to detect EV subpopulations expressing low-abundance markers. The technique also does not provide detailed information on vesicular cargo and cannot reliably distinguish EVs from similarly sized particles, such as lipoproteins or protein aggregates, without prior rigorous purification [93].

Transmission Electron Microscopy (TEM), on the other hand, remains a gold-standard method for confirming EV morphology. It also enables the localization of exosomal markers through immunogold labeling using antibodies against proteins such as CD9, CD63, and CD81 [3,94]. Despite its high resolution and specificity, TEM is expensive, time-consuming, and requires specialized expertise and instrumentation.

In recent years, flow cytometry platforms have advanced considerably. Instruments like the CytoFLEX Nano allow for the sensitive detection of submicron particles as small as 40 nm. This technology enables detailed immunophenotyping of EVs by detecting both surface and intracellular antigens with fluorochrome-conjugated antibodies against classical tetraspanins (CD9, CD63, CD81) and cell-origin markers such as CD41a (platelet-derived EVs) and CD3 (T cell-derived EVs) [7,8,94]. Furthermore, the use of permeabilizing agents combined with antibodies allows for the detection of intracellular molecules. The incorporation of NIST-traceable size standards, along with the FCMPASS software, has enabled the standardization of light scatter measurements using Mie theory-based calibration curves. This allows scatter data to be reported in comparable physical units (e.g., nanometers), thereby improving EV characterization and enabling more consistent assessment of EV size and concentration, particularly in instruments capable of event counting [95].

Western blotting is also commonly used to identify specific proteins in EV preparations and represents a relatively low-cost and accessible method to validate the presence of EV-associated markers. According to the International Society for Extracellular Vesicles (ISEV), proper characterization should include the detection of at least one transmembrane protein (e.g., CD9 or CD63) and one cytosolic protein (e.g., TSG101 or ALIX), as well as negative markers such as albumin or uromodulin to assess purity [10]. This technique is widely compatible with standard laboratory infrastructure, making it practical in many research settings.

Nevertheless, Western blotting has several limitations. It is semi-quantitative and has limited sensitivity, making it less effective for detecting low-abundance proteins or small EV subpopulations. It also provides no information on particle size, concentration, or heterogeneity, and cannot confirm whether the detected proteins are associated with intact EVs or are free-floating contaminants. Furthermore, it often requires relatively large protein inputs, which can be challenging when working with limited sample volumes or low EV yields [10].

Simultaneously, nucleic acid analysis of EVs is routinely performed using traditional methods like quantitative PCR (qPCR) and more advanced platforms such as next-generation sequencing (NGS). These techniques allow for the detection of microRNAs, other non-coding RNAs, and DNA contained within EVs. A wide range of commercially available kits has been developed for the extraction and purification of EV-derived microRNAs, enhancing both the efficiency and reproducibility of downstream analyses [92].

To ensure reproducible and high-quality results, it is essential to implement rigorous quality control, appropriate pre-analytical handling of samples, and continuous validation of methods. Moreover, adherence to standardized protocols aligned with international guidelines—such as the Minimal Information for Studies of Extracellular Vesicles (MISEV)—is strongly recommended [92]. This will support the robust implementation of EV-based diagnostics for dengue in clinical settings and future research applications.

## 12. EVs as Potential Biomarkers for Dengue Severity: Role of NS1

EVs play a crucial role in the pathophysiology of dengue, particularly in endothelial dysfunction and vascular hyperpermeability, processes that are vital for disease progression [96,97]. These vesicles not only amplify the damage directly by promoting viral spread but also serve as molecular markers of the infection’s severity. In severe cases of dengue, such as hemorrhagic fever and dengue shock syndrome, the profile of circulating EVs changes, reflecting an increase in viral load, the presence of viral proteins like NS1, and modulation of the host’s immune response [41,73]. The presence of NS1 in circulating EVs is a key marker for diagnosing and monitoring dengue infection, but additional molecular components of EVs, such as viral RNA, miRNAs, and host proteins involved in immune modulation, could also serve as valuable biomarkers.

Analyzing the specific molecular content of these EVs could provide new biomarkers for predicting the transition from mild to severe dengue and monitoring therapeutic effectiveness. Identifying biomarkers based on circulating EVs offers a powerful tool for early detection of dengue progression and for tailoring treatment strategies. The role of EVs in viral material transport, their influence on inflammatory pathways, and their contribution to viral load highlight their importance in the pathogenesis of dengue. Therapeutic strategies that block the formation or release of EVs, or interfere with their interaction with endothelial cells, could prove promising for mitigating the severe effects of DENV infection and improving clinical outcomes.

## 13. Differentially Expressed miRNAs in Dengue Patients and Potential Signaling Pathways Involved

To investigate the relationship between miRNAs and DENV infection or severity, we reanalyzed DEmiRs from an RNA sequencing dataset of the circulating microtranscriptome of dengue patients (GSE150623) [98], using the limma-voom pipeline in R.

**Figure 3:**
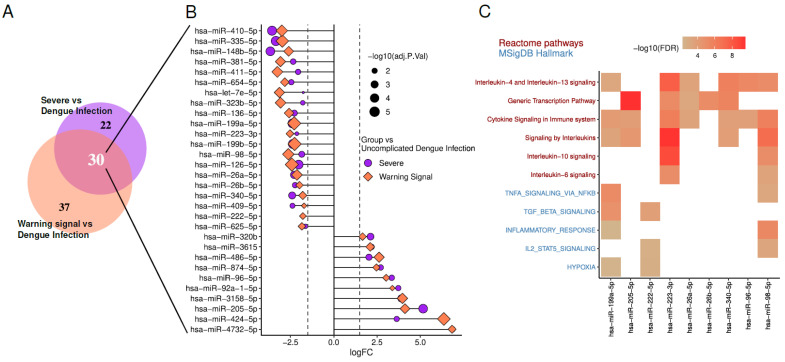
Analysis of circulating miRNAs in dengue infection (GSE150623) reveal 30 miRNAs differentially expressed (|LogFC| > 2 and Adjusted *p*-value < 0.05) mutually in severe and warning signal cases compared to dengue infection without signs. (**A**) Venn diagram of deMIRs of commom regulation between severity. (**B**) List of common deMIRs and its expression in both groups compared to uncomplicated dengue infection. (**C**) En-richment pathways of deMIRs.

We focused on DEmiRs with |LogFC| ≥ 2 and adjusted *p*-value < 0.05. In both Severe vs. Dengue Infection (DI) and Warning Sign vs. DI comparisons. Among them, 20 miRNAs are downregulated, indicating a decrease in their expression levels. In contrast, 10 miRNAs are upregulated, showing increased expression levels. Enrichment analysis of predicted targets for these DEmiRs was performed through Diana Tools miRPath v4 [14]. Enrichment terms associated with DENV manifestations are illustrated in Figure 3.

Regarding hypoxia, it has been demonstrated that the secretion and composition of exosomes are closely influenced by oxygen pressure in the microenvironment. As reviewed by Yaghoubi et al. (2020), several studies suggest that cells respond to hypoxia by altering the molecular profile of exosomes [99]. In vitro analyses further revealed that monocytes under hypoxic conditions required higher concentrations of antibodies for DENV neutralization [100]. For Stat5, members of the JAK-STAT signaling pathway play a critical role in the interferon-induced inflammatory response, driving the expression of antiviral and pro-inflammatory proteins during DENV infection [101]. Interestingly, the inhibition of Janus Kinase (JAK), which prevents interferon signaling, has shown therapeutic effects in experimental model of arenavirus hemorrhagic fever, suggesting potential applicability to DENV infections [102].

Concerning TNFα, blocking TNFα alone reduced plasma leakage, while co-treatment with sunitinib significantly improved the survival of DENV-infected mice without affecting viremia [103]. DENV entry via ADE has been shown to suppress the host cell’s antiviral response by inhibiting IFN-α production while inducing IL-10 expression, thereby facilitating increased viral replication. This aligns with clinical observations of higher viremia, elevated IL-10 levels, and reduced IFN levels in patients with severe dengue [104]. Additionally, interleukins 4, 6, 10, and 13 were found to be significantly elevated in EVs derived from patients with severe dengue [64].

These findings underscore the importance of miRNAs in modulating critical biological processes during DENV infection, including immune responses, cytokine regulation, and cellular signaling pathways. The observed changes in miRNA expression, coupled with their enrichment in pathways linked to hypoxia, JAK-STAT signaling, and pro-inflammatory cytokines, suggest that miRNAs may serve as potential biomarkers for disease severity and therapeutic targets.

## 14. Potential Therapeutic Strategies Based on Current Knowledge of EVs

Extracellular vesicles, including exosomes, microvesicles, and apoptotic bodies, have gained significant attention for their therapeutic potential. These vesicles naturally facilitate intercellular communication by transferring bioactive molecules such as proteins, RNAs, and lipids. Their ability to influence various biological processes like immune modulation, tissue regeneration, and disease progression has led to the exploration of several therapeutic strategies.

One promising approach involves the use of extracellular vesicles (EVs) as drug delivery vehicles. Their ability to encapsulate therapeutic cargo and protect it from degradation, along with their inherent biocompatibility and capacity to cross biological barriers, makes them ideal candidates for targeted drug delivery. EVs can be engineered to carry chemotherapeutic agents, gene-editing tools, or immune-modulating molecules, demonstrating significant potential in the treatment of cancer, genetic disorders, autoimmune diseases, and infectious diseases.

Extracellular vesicles have emerged as versatile tools in therapeutic development due to their ability to modulate immune responses, promote tissue regeneration, and serve as non-invasive biomarkers. For instance, EVs derived from regulatory T cells or mesenchymal stem cells can suppress inflammation and enhance immune tolerance, making them valuable in the treatment of autoimmune diseases. Conversely, tumor-derived EVs can be engineered to enhance anti-tumor immune responses. In regenerative medicine, EVs facilitate tissue repair by delivering growth factors, cytokines, and mRNAs that stimulate cell proliferation and modulate inflammation particularly mesenchymal stem cell-derived EVs, which have been studied for applications in cardiovascular disease, stroke, and bone healing. Additionally, the molecular cargo of EVs reflects the physiological state of their cells of origin, making them promising candidates as biomarkers for early diagnosis and disease monitoring in conditions such as cancer. Their surface can also be modified with ligands (e.g., antibodies or peptides) to enable precise therapeutic targeting, thereby minimizing off-target effects and improving treatment efficacy.

In the context of dengue virus infection, EVs, mainly exosomes, offer novel therapeutic opportunities. EVs derived from dendritic cells have been shown to activate CD4+ T cells and modulate Th1 or Th2 responses depending on their size. Antigenic stimulation enhances the incorporation of MHC class II molecules into these vesicles, and their uptake by recipient dendritic cells further amplifies T cell activation and immune modulation. Building on these findings, the targeted loading of dengue virus antigens into exosomes may represent a promising strategy to stimulate both CD4+ and CD8+ T cell responses, thereby strengthening the adaptive immune response. This approach could improve antigen presentation and guide T cell functional polarization, offering potential applications in vaccine development or immunotherapeutic interventions for dengue and other viral infections.

Moreover, EVs can be engineered to deliver antisense oligonucleotides that inhibit microRNAs essential for viral replication. A precedent for this strategy is provided by Santaris Pharma, which developed an anti-miR compound targeting miR-122 a microRNA critical for hepatitis C virus replication. This compound demonstrated safety and antiviral efficacy in phase II clinical trials. Adapting such approaches to dengue could enable the development of innovative antiviral therapies using antisense oligonucleotides, provided that their safety, pharmacodynamics, and pharmacokinetics are thoroughly validated through dose-escalation studies.

## 15. Concluding Remarks

An expanding body of evidence underscores the critical role of extracellular vesicles (EVs) in the pathogenesis and progression of dengue virus (DENV) infection. EVs actively participate in viral dissemination, immune modulation, and endothelial dysfunction key features of dengue pathophysiology, especially in severe cases such as dengue hemorrhagic fever and dengue shock syndrome.

In dengue patients, circulating EVs contribute to immune dysregulation by transporting immunosuppressive molecules, including checkpoint inhibitors such as PD-L1 and anti-inflammatory cytokines like IL-6 and IL-10. These components suppress antiviral immune responses and facilitate viral persistence. Furthermore, EVs carry microRNAs (miRNAs) such as miR-146a and miR-21, which are known to reprogram host cell signaling pathways, promoting immune evasion while exacerbating inflammatory cascades. In severe disease, EV-associated miRNAs and viral proteins, particularly NS1 compromise endothelial integrity through Toll-like receptor (TLR) activation and reactive oxygen species (ROS) signaling, leading to vascular hyperpermeability and plasma leakage.

Beyond their pathogenic role, EVs hold great promise as diagnostic and prognostic biomarkers. Elevated EV concentrations and their molecular cargo, including PD-L1, miR-21, and miR-126-5p, have been correlated with higher viral loads and poor clinical outcomes. These findings support their potential as tools for early detection and risk stratification in dengue.

EVs also represent a promising platform for therapeutic development. Their biocompatibility, capacity for targeted delivery, and ability to cross biological barriers make them suitable for delivering antiviral agents, immunomodulatory molecules, or antisense oligonucleotides designed to suppress viral replication. For instance, targeted delivery of dengue virus antigens via exosomes may enhance CD4+ and CD8+ T cell responses, improving adaptive immunity. Likewise, miRNA-based interventions such as antisense strategies modeled after those used in hepatitis C virus could be adapted for dengue therapy, pending comprehensive pharmacological validation.

Ongoing technological advancements further enhance the feasibility of EV-based applications. The development of microfluidic devices, biosensors, and point-of-care diagnostic platforms is paving the way for rapid, cost-effective detection of EV-associated biomarkers, particularly in resource-limited settings. Simultaneously, progress in standardizing EV isolation and miRNA profiling will strengthen their clinical utility.

## Data Availability

Bioinformatics analyses performed in this article are available upon request. Inquiries should be directed to jshenao@uceva.edu.co.
